# Comparison of 16S and whole genome dog microbiomes using machine learning

**DOI:** 10.1186/s13040-021-00270-x

**Published:** 2021-08-21

**Authors:** Scott Lewis, Andrea Nash, Qinghong Li, Tae-Hyuk Ahn

**Affiliations:** 1 Program in Bioinformatics and Computational Biology, Saint Louis University, St. Louis, MO USA; 2 Nestlé Purina Research, St. Louis, MO USA; 3 Department of Computer Science, Saint Louis University, St. Louis, MO USA

**Keywords:** Metagenomics, Microbiome, Machine learning, Classification, Supervised learning, Diabetes, Dog, Diet, Whole genome shotgun, 16S amplicon

## Abstract

**Background:**

Recent advances in sequencing technologies have driven studies identifying the microbiome as a key regulator of overall health and disease in the host. Both 16S amplicon and whole genome shotgun sequencing technologies are currently being used to investigate this relationship, however, the choice of sequencing technology often depends on the nature and experimental design of the study. In principle, the outputs rendered by analysis pipelines are heavily influenced by the data used as input; it is then important to consider that the genomic features produced by different sequencing technologies may emphasize different results.

**Results:**

In this work, we use public 16S amplicon and whole genome shotgun sequencing (WGS) data from the same dogs to investigate the relationship between sequencing technology and the captured gut metagenomic landscape in dogs. In our analyses, we compare the taxonomic resolution at the species and phyla levels and benchmark 12 classification algorithms in their ability to accurately identify host phenotype using only taxonomic relative abundance information from 16S and WGS datasets with identical study designs. Our best performing model, a random forest trained by the WGS dataset, identified a species (*Bacteroides coprocola)* that predominantly contributes to the abundance of *leuB*, a gene involved in branched chain amino acid biosynthesis; a risk factor for glucose intolerance, insulin resistance, and type 2 diabetes. This trend was not conserved when we trained the model using 16S sequencing profiles from the same dogs.

**Conclusions:**

Our results indicate that WGS sequencing of dog microbiomes detects a greater taxonomic diversity than 16S sequencing of the same dogs at the species level and with respect to four gut-enriched phyla levels. This difference in detection does not significantly impact the performance metrics of machine learning algorithms after down-sampling. Although the important features extracted from our best performing model are not conserved between the two technologies, the important features extracted from either instance indicate the utility of machine learning algorithms in identifying biologically meaningful relationships between the host and microbiome community members. In conclusion, this work provides the first systematic machine learning comparison of dog 16S and WGS microbiomes derived from identical study designs.

**Supplementary Information:**

The online version contains supplementary material available at 10.1186/s13040-021-00270-x.

## Background

The gut microbiome plays a significant role in maintaining the overall health of the host, nutrient absorption, as well as the overall metabolic homeostasis [1]. Further, dysfunction in the gut microbiome has been associated with host diseases including inflammatory bowel disease, obesity, and type 2 diabetes in humans [2–5]. A diverse landscape of bacteria is commonly found in the gut microbiomes of healthy individuals, but variations in strain and relative abundance make important functional distinctions. Some members may be commensal in a healthy microbiome, while others have been linked to inflammatory conditions such as rheumatoid arthritis [6]. Under certain conditions of omnivorous diets, a few strains have been linked to the enrichment of *leuB*, a gene involved in branched chain amino acid biosynthesis, which is a risk factor for glucose intolerance and type 2 diabetes [7, 8]. It has recently been shown that the composition of dog microbiomes shares significant overlap with human microbiomes when compared to the observed overlap in other animal models [9, 10]. However, metagenomic studies have also shown that dogs’ intestinal microbiota and its modification by prebiotics, probiotics, and synbiotics reveal that the dysbiosis network underlying inflammatory bowel disease in dogs differs from that in humans [11, 12], establishing a need for further research to understand the microbial communities present in dog microbiomes.

The most common technology used to analyze microbial composition is 16S rRNA gene amplicon analysis, which amplifies a 16S rRNA region with PCR and primers that recognize highly conserved gene regions. 16S amplicon sequencing (16S) has advantages due to its low cost and well-established analysis pipelines, but has limited resolution and low sensitivity compared to whole genome shotgun sequencing [13, 14]. 16S annotation relies on the association of a specific 16S rRNA gene with a taxon; these associations are defined as operational taxonomic units (OTUs). Because OTUs are most commonly analyzed at the phyla or genera resolution, 16S technologies have a limited scope in analyzing microbial communities at the species and strain levels. Recently, whole genome shotgun sequencing (WGS) has been adopted to offer several advantages by extending the range of capture in species and even strain-level resolution, and to other microbes including viruses [15]. However, with an increased rate of detection, it is necessary to thoroughly consider the potential for overconfidence and false positives during interpretation. With ample coverage provided, these methods together provide a diverse picture of the microbiome.

Indeed, both technologies are currently being used to study the microbial landscape of the gut and have been evaluated for their inherent strengths and weaknesses [16]. The choice of shotgun or 16S approaches usually depends on the nature of the study. 16S is well suited for analysis of a large number of samples (multiple patients, longitudinal studies, etc.) due to its low cost per sample, but may offer limited taxonomic and functional resolution compared to shotgun metagenomics. Shotgun metagenomics offers a greater potential for higher resolution by enhanced detection of bacterial species and even viruses, but data analysis pipelines are not well established compared to 16S, which can lead to inaccurate detection of microbes. To address this technology gap, new advanced sequencing techniques are continuing to be developed and evaluated including shallow shotgun sequencing [17].

The continued advancement in sequencing technologies has generated large quantities of data. In metagenomics a variety of computational tools have been developed to facilitate the interrogation of these large-volumes of complex metagenomic data, making use of cutting-edge computing hardware and flexible infrastructures [18]. QIIME 2 has been used prominently in the analysis of 16S amplicon sequencing data and has recently extended spatiotemporal informatics into other technologies such as metabolomics and shotgun metagenomics [19]. MetaPhlAn2 was developed initially for the analysis of increasingly large metagenomic datasets such as the Human Microbiome Project, using clade-specific marker genes to classify microbial reads with high speed and efficiency and has become a common component in whole genome shotgun metagenomic analysis workflows [20]. A variety of tools including taxonomic classification tools with nucleotide level resolution, *de novo* assemblers, strain-level profilers, and functional analysis tools are also intensively used in metagenomics research [21–26]. Moreover, machine learning techniques are now being deployed to analyze metagenomic samples and hold a great promise in extracting complex microbial patterns from samples [27, 28]. These patterns in microbial communities have the potential to deliver valuable insights in the context of studying the impact of the microbes in human and pet health and diseases. Here, we employ the QIIME 2 [19] and MetaPhlAn2 [20] workflows to profile the composition of overweight (OW) and lean (LN) dog microbiomes fed high protein low carbohydrate (HPLC), low protein high carbohydrate (LPHC), and a baseline diet. There have been few studies directly comparing the resolution and information gain inherent within the two technologies [29], and our work would be the first effort to directly compare the utility of 16S and WGS data from the same dogs in retrieving the most informative flora features from accurate phenotypic classification. We identify a disparity in the number of detected taxonomies between the two sequencing technologies and identify specific strain-level characteristics detailing interesting features within the WGS data. In order to understand how the specific properties of these two technologies may impact the profile of the dog microbiome and its influence in phenotypic response to different dietary conditions, we examined multiple machine learning models to find unique features that characterize the dysbiosis of dogs.

## Results

### Analysis of detected bacterial taxonomy

**Fig. 1:**
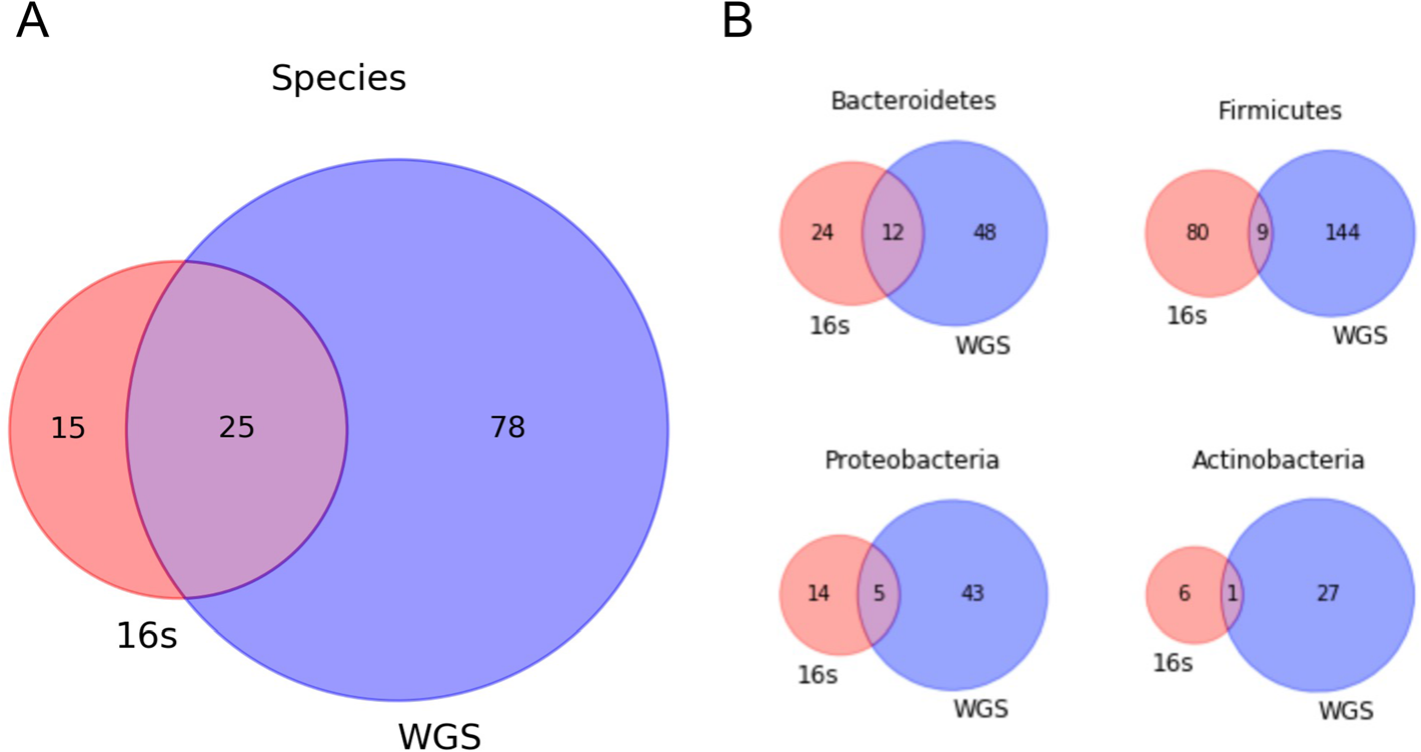
**A**. Comparing the sensitivity of microbial detection between 16S amplicon and WGS sequencing technologies reveals whole genome sequencing captures considerably more unique members at the species level. **B**. The detected members of each predominant phylogenetic group at any level. The highest membership across the four predominant phyla in 16S (red) and WGS (blue) was identified in WGS

We first compared the detection rate of 16S and WGS technologies at all available taxonomic levels to determine how these similarities and differences influence the presence of dominant phyla within the gut microbiome of dogs. For all direct comparisons between whole genome shotgun and 16S amplicon data sets, the WGS dataset was downsampled to 1 sequenced run per sample to have the same sample size of 16S dataset. We analyzed the effects of different technologies on the complexity of microbial communities by comparing the relative abundance of detected bacterial species belonging to one of 4 predominant phyla in the microbiome (*Bacteroidetes*, *Firmicutes*, *Proteobacteria*, and *Actinobacteria*). In our primary analysis of 16S microbiomes, we employed a naïve bayes model pretrained with the Greengenes 99% reference database to classify taxonomies using amplicon sequence variants (ASVs) dereplicated and filtered by DADA2, identifying 40 unique species. For the WGS analysis, we selected MetaPhlAn2 and 103 species were identified from it. By comparing species detected by 16S rRNA amplicon sequences and WGS reads, we identified species that were present in both or only one of the two technologies. The observed overlap of detected species between the two technologies was 10.2%, where 78 species were unique to WGS and 15 were detected exclusively by 16S amplicon sequencing (Fig. 1A). We further investigated the shared taxonomies identified between the two technologies with respect to four predominant phyla that are commonplace in the gut microbiome. Both methods shared 14.3% of the *Bacteroidetes*, while 2.9% of the *Actinobacteria* species were overlapping between the technologies. Additionally, WGS and 16S taxonomies shared 8.1% and 3.9% of detected phyla belonging to the *Proteobacteria* and *Firmicutes* groups, respectively (Fig. 1B). Overall, WGS shows a higher detection of taxonomies at the species level and at any level across four predominating phyla groups. We also observed a similar difference in detection rates when comparing 16S to WGS at full coverage (Supplemental Figure S1).

In order to evaluate alternative methods for assigning taxonomies in 16S and WGS analyses, we also examined different taxonomic assignment approaches. For the 16S analysis, we performed OTU dereplication and clustering with VSEARCH followed by BLAST with a minimum conservation threshold of 90% using both Greengenes and SILVA 99% references (Supplementary Table 1A). With this approach, we identified 25 unique species using the Greengenes reference and 28 unique species using the SILVA reference. The combined results from both references yielded 27 of the 40 unique species identified in our primary analysis, underscoring greater species detection using the ASV-based approach while limiting false positives through the error model fitted by DADA2. For the alternative WGS taxonomy assignment, we have run Kraken 2 [26], which identified 3247 unique members at the species level. Compared to the MetaPhlAn2 results reported in our primary analysis which identified 103 unique species the species detection rate by Kraken 2 is nearly 30 times greater, thus Kraken 2 provides high sensitivity at the risk of low specificity and a greater rate of false positives. In all primary analyses, we used MetaPhlAn2 results for post taxonomy identification to provide a more conservative estimate of taxonomy assignment.

### Functional gene orthologs

**Fig. 2:**
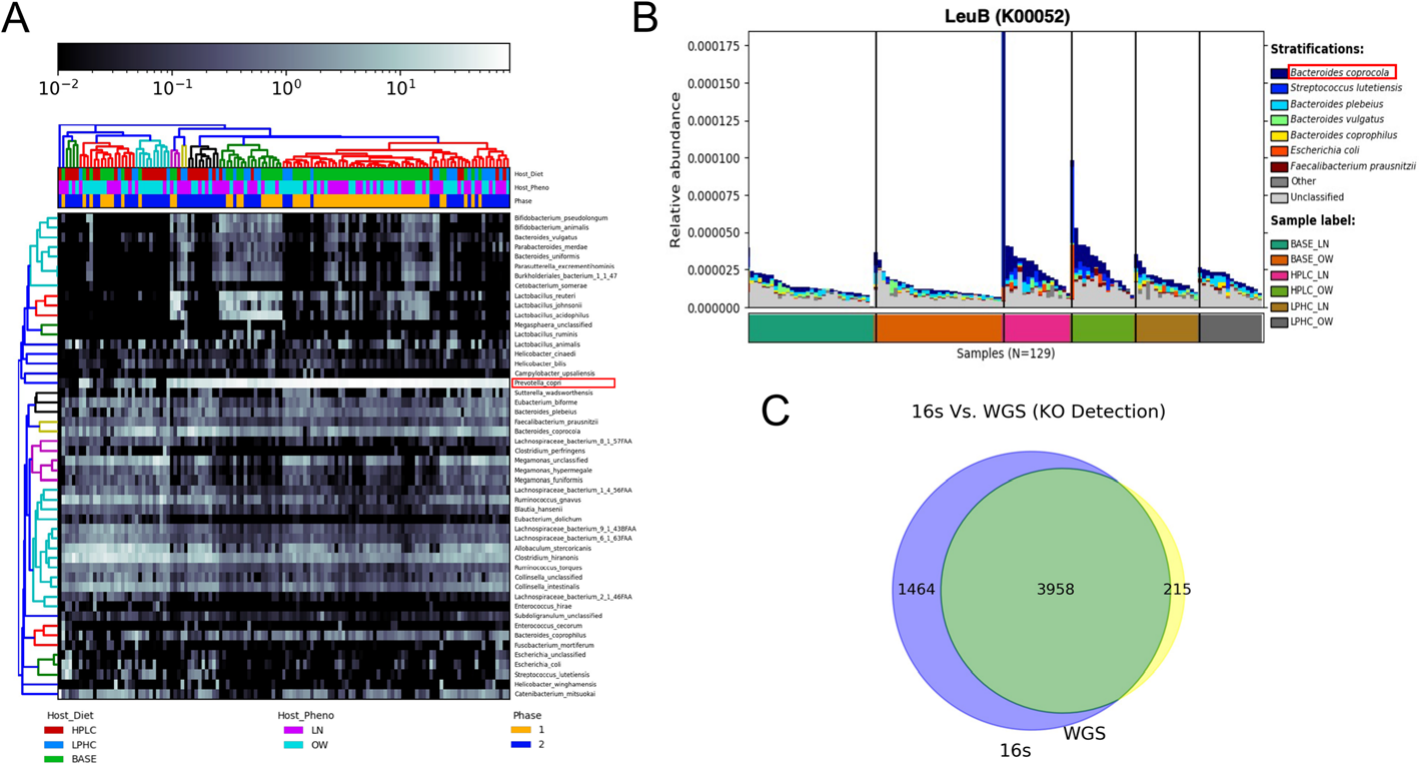
**A**. *Bacteroides coprocola* is more abundant in carbohydrate-rich diets. The relative abundance is greatest in base diet conditions and significantly lower in the microbiomes of high-protein diets (p < 0.05, Kruskal-Wallis H). **B**. The striated output of individual contributions to *leuB* abundance in the different diet-phenotype groups (baseline diet (BASE), high protein low carbohydrate (HPLC) and low protein, high carbohydrate (LPHC) with overweight (OW) and lean (LN) phenotypes) reveals *leuB* is the most abundant in HPLC diet groups and *B. coprocola* is largely contributing to this abundance. **C**. Comparing the detected gene orthologues between WGS (yellow) and 16S (blue) data sets indicates the majority are conserved between the two technologies (green)

In order to profile the orthologous gene groups and pathways present in 16S and WGS dog metagenomic data, we identified KEGG orthologs (KOs) used PICRUSt2 [30] and HUMAnN2 [31] respectively. Among the detected species, we tested for significance of differential abundance between dietary groups (Kruskal-Wallis H-test) and identified a reduced abundance of *Prevotella copri* in WGS samples of dog microbiomes fed the HPLC diet (Fig. 2A). We also report the abundance of *leuB* is significantly enriched in the microbiomes of dogs that were fed the HPLC diet and is strongly associated with *B. coprocola*, rather than *P. copri.* (Fig. 2B). Bray-Curtis Dissimilarity of detected WGS microbial communities and gene families projected by metric multidimensional scaling shows many of the samples with high *B. coprocola* and *leuB* abundance originate from HPLC or LPHC diets. Additionally, these samples show dissimilarities from those with lower *B. coprocola* and *leuB* relative abundance (Supplemental Figures S2 and S3).

### Comparison of gene orthologues

Comparing the gene orthologs from detected 16S and WGS taxonomies, we characterized a remarkable overlap (Fig. 2C). This was a somewhat surprising result, considering functional annotations enabled for 16S amplicon sequencing are inference based, while functional annotations for WGS sequences use gene-level features directly. Nevertheless, ubiquitous or overlapping annotations may be primarily driving this observation, which is a common issue in many gene ontology databases. While many of the same gene orthologs were shared between the microbiomes captured by 16S and WGS, the differential abundances of many of these gene orthologs were not mutually significant between the data sets (data not shown). Notably, *leuB* was only significantly enriched in the WGS microbiome profiles of samples in the HPLC diet groups (Fig. 2B). With respect to the stratified WGS relative abundance, the specific contributions to *leuB* relative abundance were made predominantly by members of the *Bacteroides* genus, with the exception of the second most abundant species, *Streptococcus lutetiensis*. Overall, *B. coprocola* was the most abundant species contributing to *leuB* enrichment in HPLC microbiomes.

### Strain-level analysis

**Fig. 3:**
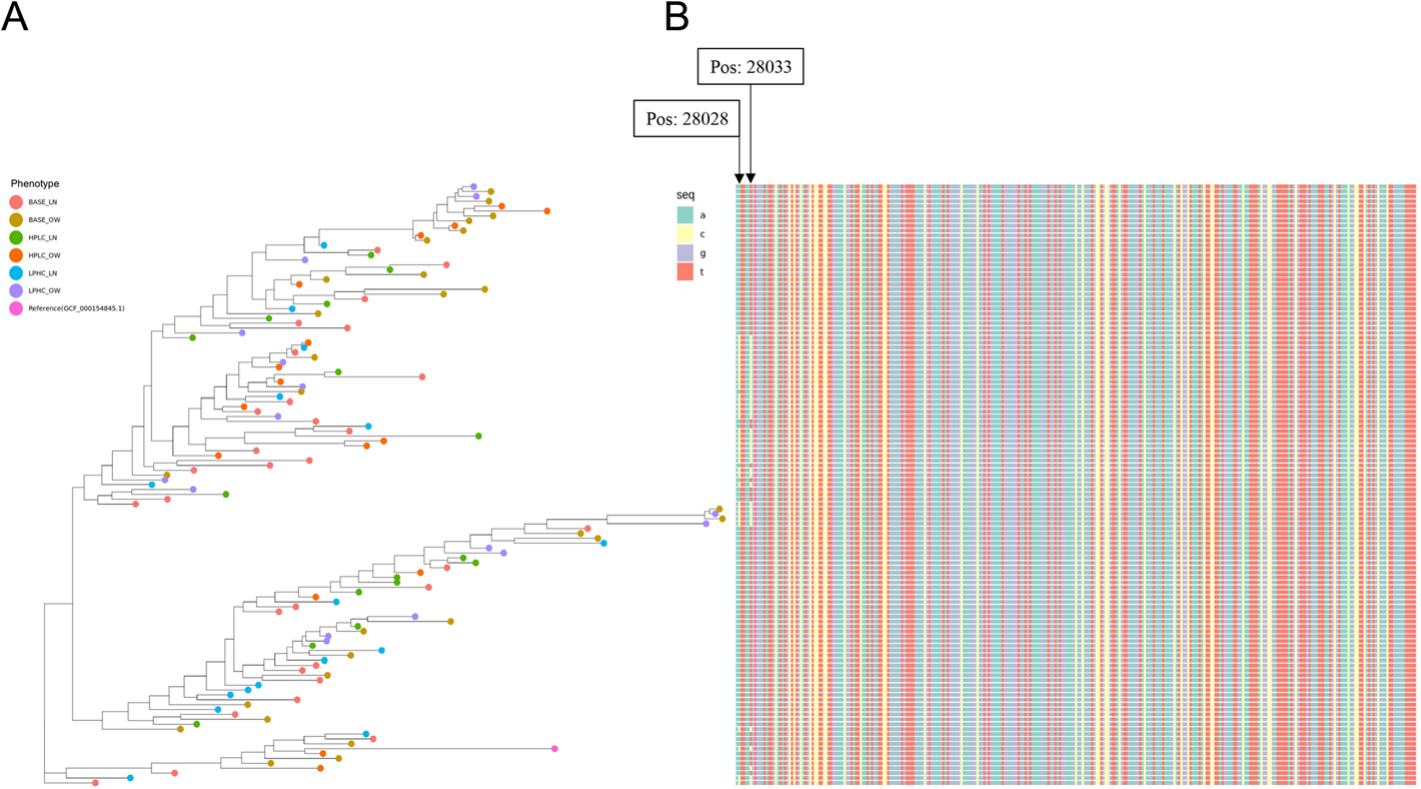
**A**. The phylogenetic tree generated by RAxML (Randomized Axelerated Maximum Likelihood) of *B. coprocola* genomes present in all WGS samples. The reference genome (GCF_000154845.1) is shown in pink. Strains between samples labelled by phenotype (baseline diet (BASE), high protein, low carbohydrate (HPLC) and low protein, high carbohydrate (LPHC) with overweight (OW) and lean (LN) phenotypes) show minimal preferential clustering. **B**. The corresponding multiple sequence alignment represents the *leuB* gene sequence for each member of the phylogenetic tree. The sequence is largely conserved across all clusters with the exception of SNPs at positions 28028 and 28033, suggesting the increase in leuB relative abundance in HPLC diets is driven by the abundance of *B. Coprocola*

**Fig. 4:**
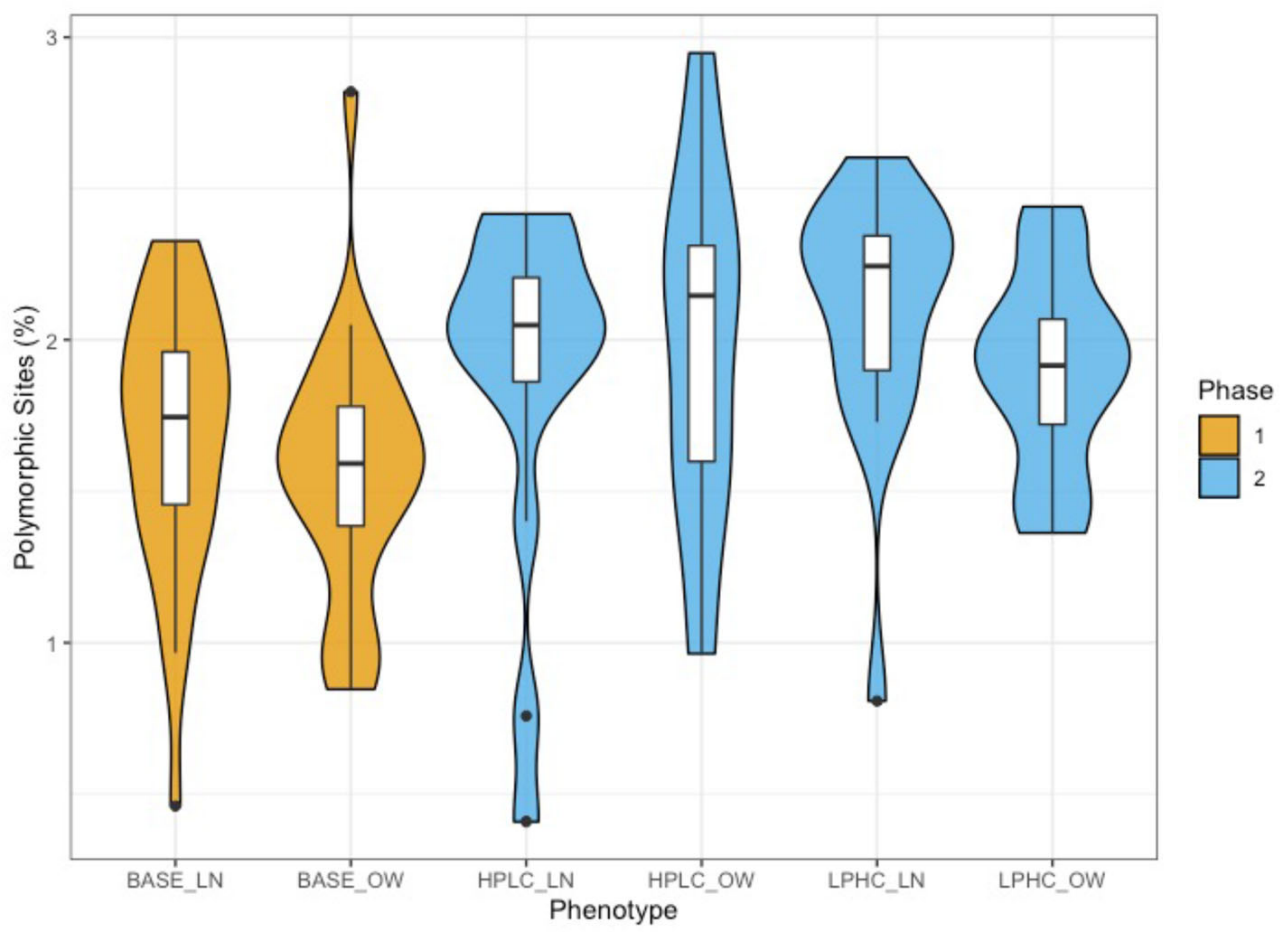
The mean and quantile distribution of the percent polymorphic sites for each sample with respect to the diet-weight phenotype. On average, samples from phase 2 that were fed the HPLC or LPHC diets showed a significantly (p < 0.01) higher percentage of polymorphic sites than samples from dogs fed the base diet in phase 1. Significance was tested using the Wilcoxon rank sum test with Bonferroni correction for multiple comparisons

Analysis of strain-level cladistics revealed 200 distinct markers for *B. coprocola* present in 123 samples after gap corrective filtering. We constructed a phylogenetic tree from these sequences along with a reference genome from the RefSeq database (GCF__000154845.1) using Randomized Axelerated Maximum Likelihood (RAxML) with and without bootstrapping. The strains identified across samples from all phenotypes showed minimal segregation between clusters with respect to phenotype, suggesting that there are minimal differences in *B. coprocola* at the strain-level in the microbiomes of overweight or lean dogs fed the base, HPLC, or LPHC diets (Fig. 3A). We further investigated the single nucleotide polymorphisms (SNPs) present in the region of the multiple sequence alignment composed of the species-specific marker sequences that corresponded to *leuB* (NZ_DS981457.1) (Fig. 3B). With the exception of SNPs present at positions 28028 and 28033, the sequences encoding *leuB* are largely conserved between strains. We also report the mean and quantile distribution of the percent polymorphic sites of identified strains for all body weight groups (Fig. 4). We note that on average, dog samples from phase 2 diet groups had higher percentages of polymorphic sites than samples from dogs that were fed the base diet. We tested this difference with a Wilcoxon rank sum test with continuity correction and found the difference between ranks of the phase 1 and phase 2 diets groups to be statistically significant (p < 0.01). Overall, the results of these analyses suggest that there is a significant enrichment of polymorphic sites in phase 2 diets, although the attributes of significant diet specific abundance enrichment differ in taxonomic composition.

### Phenotype classification

**Fig. 5:**
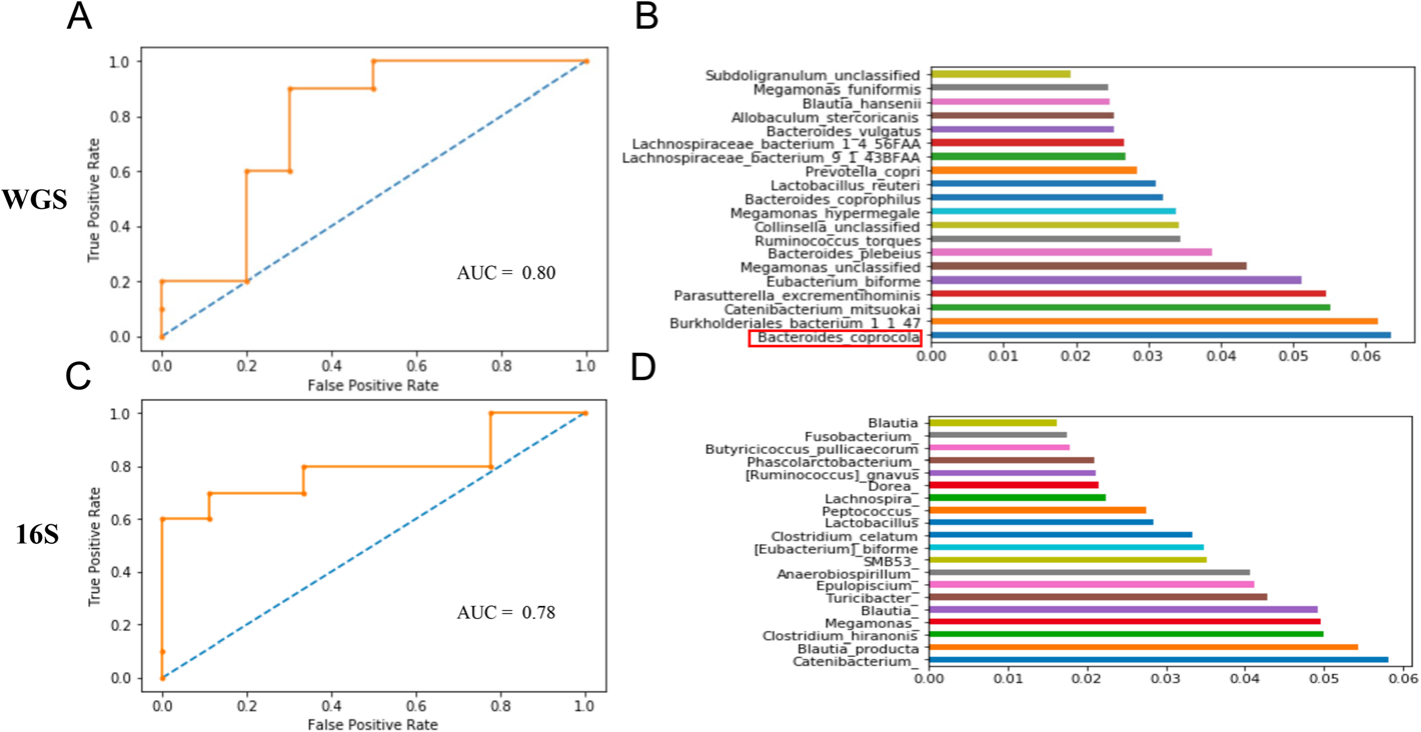
The ROC curve of the random forest model’s performance and most important features used in classifying weight phenotype for WGS (**A, B**) and 16S (**C, D**). The importance score for a feature is the fraction of samples across all trees that traverse a node and split on that feature. Notably, *B. coprocola* was the most important feature for classifying the weight phenotype in WGS samples

For phenotypic classification, we used samples from the phase 1 cohort that were fed the base diet for training with 30% of these samples set aside to be used as a validation set while developing each classification model. We initially used the multiple sequencing runs available in the WGS data set as individual samples for training. After an exhaustive grid search for optimal hyperparameters, our random forest model produced an overall accuracy score of 97% at the file-level (Table 1). While this method rendered a high accuracy score, this was likely due to the model recognizing overlapping run profiles derived from the same dog sample in the validation set. To remove the overlap between training and validation sets, we pooled runs to their respective samples before splitting the profiles into training and validation sets. In total, we evaluated the performance of 12 different machine learning classification algorithms in classifying the weight phenotype (overweight or lean) of the host, using only relative abundances of genus and species level taxonomy from QIIME 2 and MetPhlnAn2 output as training. The overall percent accuracy, log loss, and area under the curve (AUC) of selected models is reported in Table 2. Out of all models tested, the random forest algorithm achieved the best performance across all metrics in classifying the weight phenotype of both WGS and 16S amplicon taxonomic profiles (Supplemental Figure S4). We also evaluated the top performing random forest model on 2 permutations of the phase 2 cohort resulting from the HPLC and LPHC dietary groups in both the 16S and WGS data sets to investigate taxonomies that contributed the most information gain during classification. One advantage of the random forest model is that important features that were used to split samples traversing a node can be extracted. We leveraged this characteristic of tree-based learning methods to extract the features that were most important during classification. Specifically, we looked at which genera or species were most important in classifying the weight phenotype of dogs fed the HPLC diet (Fig. 5). In addition to *B. coprocola* being the primary driver for *leuB* enrichment in HPLC diets, it was the most important feature for classifying weight phenotype in WGS samples (Fig. 5B). With respect to 16S HPLC samples, the *Megamonas* and *Lachnospira* genera were among the most important features for classifying taxonomic profiles from overweight and lean dogs (Fig. 5D). We note that the most important features derived from whole genome and 16S amplicon sequencing weight profile classifications had little overlap, although both collections of important features support the influence of specific microbiome profiles on host body fat composition previously described in the literature.

**Table 1:**
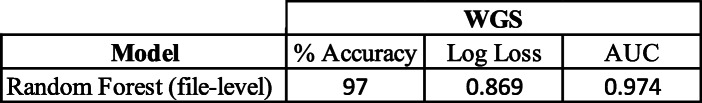
Classification metrics for the top performing random forest model with optimization at the file-level, without merging technical replicates into respective sample bins

**Table 2:**
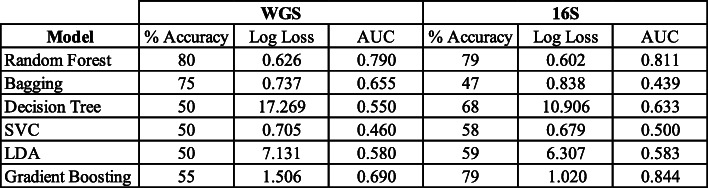
Classification metrics of model performance classifying samples sequenced in their respective technology. The random forest model achieved the highest percent accuracy with minimal log loss and maximal AUC in classifying taxonomic profiles derived from either sequencing technology

### Discussion

In this study, we have compared the resolution of taxonomic profiles rendered by 16S amplicon and WGS technologies. By comparing the microbiomes of samples sequenced with these technologies, we have shown that the resolution of functional taxonomy groups detected by whole genome shotgun sequencing is greater at the species level as well as in members at any level belonging to four predominant phyla. This difference in resolution may have the potential to inform future metagenome-scale studies. *Megamonas* and *Lachnospira* genera were among the most important features for classifying taxonomic profiles from overweight and lean dogs in 16S and WGS samples, which is supported by previous studies that show these genera are associated with obesity and metabolic disorders [32]. From 16S amplicon sequenced samples, the two most important genera for weight phenotype classification were *Blautia* (*Blautia producta*) and *Catinibacterium*. Little is known about the role of the *Catinibacterium* genus in regulating fat composition in the host, however previous findings suggest that the presence of the *Blautia* genus in adult gut microbiomes is associated with visceral fat accumulation [33]. Interestingly, the majority of the most important features for classifying 16S samples were genera while the most important features for classifying WGS samples were detected at the species level.

The annotation of orthologous groups is essential in comparative genomics and is used to model a number of applications in disease, nutrition, and aging-related studies. In the context of microbiome communities, gene orthologs provide insight into functional pathways. However, the sensitivity, specificity, and resolution of orthologous gene sequence profiles at different taxonomic levels has the potential to influence these pathways. Although we report differences in the captured metagenomic landscape between the two technologies, we observed the number of reported orthologous genes is comparable between the two technologies. Although these results indicate many of the same gene ortholog groups may be conserved between the two sequencing methods, there may be species-level insights that are better assessed with WGS sequencing technologies, as demonstrated by the unique significant enrichment of *leuB* detected in the WGS taxonomy data.

It has previously been shown in human microbiomes that strains of *P. copri* regulate *leuB* relative abundance differently, with possible strain-specific selection in omnivorous diets that are higher in protein and branched chain amino acid content [7]. We found that in dogs, *P. copri* abundance is influenced by diet, and is specifically enriched in diets that are higher in carbohydrates, which contrasts previous findings in human samples. Further, a different bacterium (*B. coprocola*) is predominantly contributing to differences in *leuB* levels between diet groups instead. Strain-level analysis of WGS data revealed minimal SNP sites between *leuB* encoding sequences in different strains of *B. coprocola*, suggesting the enrichment of *leuB* in HPLC diets is likely driven by abundance rather than the strain specific differences in this gene. In addition, these results indicate that there may be a positive selection mechanism leading to increased *leuB* relative abundance when available nutrient composition is high in protein and low in carbohydrate content. Overall, this mechanism has the potential to promote insulin resistance in a different way than what has previously been observed in human samples. These results suggest that diet remains an important factor for the regulation of leucine biosynthesis by the microbiome, however the bacteria that are predominantly contributing to this process in dogs show a distinct pattern from what has previously been observed in humans. Further comparison of the average percentage of polymorphic sites between different diet-weight groups revealed that the most significant difference existed between phase 1 samples from dogs that were fed the base diet, and phase 2 samples from dogs that were fed the HPLC or LPHC diets. Interestingly, phase 2 samples had a significantly higher percentage of polymorphic sites on average. This significant difference in polymorphic sites indicates a possible strain-level diversification by diet. Although phylogenetic analysis revealed few differences in the aligned *leuB* sequence from *B. coprocola* genomes between samples across all diet and weight conditions, there may be other community members that show specific genetic diversification as a function of dietary change that could be of interest in future studies.

## Conclusions

Machine learning has proven to be a powerful tool for extracting useful patterns from large and complex data sets. The algorithms we evaluated in this study achieved highly variable accuracy in all metrics while the random forest, our best performing model, identified key functional taxonomies that support biologically meaningful insights into regulatory mechanisms driven by microbiome compositions specific to the diet and weight phenotypes of host samples. Moreover, the support for feature extraction by supervised learning methods allowed for the initial discovery of *B. coprocola* as an important feature that distinguishes the microbial composition of overweight and lean dog phenotypes in WGS samples, and *Megamonas* in 16S samples. While predictive taxonomies identified in both technologies provided biologically meaningful insights, these taxonomies were mutually exclusive in many instances, underscoring fundamentally different potential applications for 16S and WGS. The 16S amplicon sequencing method provides a robust, but limited modality for investigating the taxonomic diversity of bacterial communities through reference-based annotations and well-established analysis pipelines. A primary limitation of the 16S amplicon method is that it captures a limited region of the bacterial genome, while WGS is capable of detecting broad regions of bacterial, fungal, and viral genomes. Here, we quantified a greater number of unique community members at the species level, as well as in four predominant phyla in WGS. Through the ability of WGS to detect gene enrichment within specific community members, we identified a potential mechanism for *B. coprocola* in mediating predisposition to insulin resistance and glucose intolerance associated with type 2 diabetes in response to a high-protein, low-carbohydrate diet in dogs. Thus, WGS provides enhanced detection of bacterial species and a greater utility in implicating specific community members through quantified enrichment or depletion of genetic and metabolomic elements, as well as taxonomies with undefined roles in disease pathogenesis. Deeper mechanistic insights into the link between *B. coprocola* enrichment and diet may be of great interest to future studies addressing environmental risk-factors contributing to type 2 diabetes. More broadly, this study has provided necessary insights into the strengths and weaknesses of 16S amplicon and WGS sequencing technologies. Further, the computational and statistical methods used in supervised classification algorithms will be a powerful asset to future metagenomic studies addressing biological pattern discovery and the validation of analysis pipelines.

## Materials & Methods

### Data acquisition and study design

In this study, we reanalyzed two dog microbiome data sets, 16S amplicon (NCBI Sequence Read Archive accession number SRP095473) [9] and WGS (European Nucleotide Archive accession number PRJEB20308) [10]. The data sets share a conserved treatment and sampling interval, starting with the same 64 dogs (Labrador retrievers and Beagles). Specifically, 64 overweight and lean dogs were fed in two phases: a baseline diet for 4 weeks (phase 1), followed by 4 weeks of assignment to a diet treatment group (phase 2) where dogs were fed either a high protein, low carbohydrate (HPLC) diet or a low-protein, high-carbohydrate (LPHC) diet. Fecal samples were preserved within 15 minutes of defecation and sequenced with their respective technologies at the end of each phase. Dog weight phenotype was determined by host body fat percentage at the end of each phase.

In the WGS study, each sample’s DNA was extracted and sequenced (12 runs per sample) in paired-end mode with 125 bases per read. Overall, the WGS dataset contains 1,548 paired-end files that we analyzed at the file-level (after merging paired-end reads), and the subject-level, where files were combined to the resolution of the dogs they originated from. 1.9 terabasepairs were represented across all samples, and the average number of reads per metagenome was 117 million pared-end reads. Samples from the 16S study had an average of 48,092 ± 1,772 sequences per metagenome, a median sequence length of 422 base pairs, and a range between 57 and 449 base pairs. All direct comparisons between WGS and 16S amplicon data sets where made after downsampling the WGS dataset to 1 sequenced run per sample.

### Preprocessing

Prior to the analysis, all files were inspected for quality control. Representative amplicon 16S amplicon sequence variants (ASVs) were identified within the DADA2 workflow, which has proven to be a powerful method for denoising, dereplicating, and filtering chimeric sequences, thereby identifying high quality ASVs and reducing false positives [34]. 16S forward and reverse reads were truncated at positions 288 and 220 with respect to the 3’ end of the sequence, where bases were added in the final cycles of sequencing. The 5’ end of forward reads were trimmed at position 15 as additional quality control. The error correction method employed by DADA2 was based on training from 1 million reads to ensure a robust error model used in identifying ASVs. WGS sequence quality was ensured by trimming nucleotide positions with a quality score of less than 15, removing low-quality reads (mean quality < 25), and discarding reads shorter than 80 nucleotides. For both data sets, paired-end reads were merged prior to taxonomy assignment. The WGS data set contained multiple paired alignment files for the same sample and as an initial analysis, we did not merge reads to the sample-level and instead treated each merged paired-end file as an individual sample. For all subsequent metrics reported, we used merged paired-end reads that were pooled to the sample level from multiple files of sample replicates.

### Analysis and taxonomy assignment

16S taxonomies were assigned in QIIME 2 [19] (Version: 2019.7) by a Naïve Bayes classifier pretrained on the Greengenes 99% sequence cluster identity OTU database. Taxonomies from WGS were assigned using the MetaPhlAn2 (Version: 2.7.7) [20] clade-specific marker gene database that was generated from 17,000 reference genomes. To prepare relative abundances of taxonomies for machine learning, all taxonomic profiles were filtered to include only features that were at the species level. Functional gene orthologs were assigned from the Kyoto Encyclopedia of Genes and Genomes (KEGG) [35] using PICRUSt2 [30] (Version: 2.14 beta) for 16S data and HUMAnN2 [31] for WGS data. Statistical testing for differential abundances between taxonomies and gene orthologs was performed using the Kruskal-Wallis H test, a rank-based nonparametric test.

Alternative methods for 16S taxonomy assignment were evaluated by OTU dereplication and clustering with VSEARCH followed by BLASTn with maximum accepted hits per query of 20 (using QIIME 2 option --p-maxaccepts), a 90% minimum conservation threshold (using QIIME 2 option --p-min-consensus), and a default E- value threshold of 0.001. We evaluated this approach using both SILVA, and Greengenes 99% databases in order to relate this comparison to our primary results and evaluate two of the most widely used reference databases in the field (Supplementary Table 1A). Alternative methods for WGS taxonomy were evaluated with Kraken 2 using the same raw data preprocessing previously described (Supplementary Table 1B).

To characterize strain-level composition of WGS data, we used the StrainPhlAn2 [25] (Version: 1.0) workflow with default settings. Prior to analysis, samples and clade markers were filtered by removing consensus markers with a rate of ambiguous nucleotides greater than 20% and clades for which less than 80% of markers could be identified. Only clades supported by two or more samples were retained. Further, only samples containing full clade marker sequences with a percentage of gaps less than 20% were used for multiple alignment and phylogenetic tree construction. Phylogenetic trees were visualized using the ggtree package [36] in R. The filtered marker sequences from each sample were used as input to construct phylogenetic trees using the Randomized Axelerated Maximum Likelihood (RAxML) algorithm, with and without bootstrapping (10 iterations). The alignment of these sequences was accomplished using MUSCLE [37] (Version: 3.8.31).

Assessments of alpha and beta diversity in microbial communities were accomplished using the QIIME 2 [19] (Version: 2019.7) workflow and PhyloSeq [38] (Version: 1.32.0) in R using Faith's PD and the Bray-Curtis Dissimilarity index, respectively.

### Classification algorithms

We evaluated the performance of 12 different machine learning algorithms to classify the weight phenotype (overweight or lean) of the host, using the relative abundance of genus and species level taxonomies from QIIME 2 and MetPhlnAn2 results as training. Those machine learning models were developed through scikit-learn [39] (Version: 0.21.3), with exhaustive grid search to discover optimal hyperparameters. We chose to use a decision tree as a base model as well as a random forest (an ensemble of decision trees) to avoid overfitting. We later make use of these methods’ support for extracting important features used for classification, a characteristic of tree-based learning methods. We also evaluated the potential of gradient boosting, which builds on an additive model in a forward stage-wise fashion, allowing for the optimization of arbitrary differentiable loss functions. For this model, we used deviance as the loss parameter for classification with probabilistic outputs. Because our classification problem was binomial (overweight or lean), our gradient boosting model uses a single regression tree to fit at each additive stage to the negative regression of the binomial deviance loss function.

The support vector machine (SVM) model was optimized by evaluating the optimal combinations of hyperparameters for the starting kernel of a radial basis function. These parameters include *C*, the regularization parameter where the strength of regularization is inversely proportional to *C*. A large value of *C* will make the decision boundary for classification more rigid in order to accommodate the specific characteristics of the training data. We also assessed different values of γ, the influence that a single training example will have on the overall classification, where for higher values of γ samples must be closer to be affected. Our final values for our SVM classifier were *C* = 0.001 and γ = 0.001, using the radial basis function kernel. These parameters have the advantage of maintaining a model that generalizes well to new data without overfitting to any overly discriminatory subsets present within the training set.

Linear discriminant analysis (LDA) is another useful model for reducing the dimensionality of input classes, by projecting to the most descriptive directionalities. Our LDA model used a singular value decomposition (SVD) solver, due to the large number of features in the microbiome profiles used as training. The SVD solver does not compute a covariance matrix, therefore our model made no assumptions about the covariance profile when fitting gaussian density to each class.

## Supplementary Information

**Additional file 1: Supplemental Table 1**: Alternate taxonomic assignment approaches for 16S and WGS datasets. A. 16S species and phyla identified by dereplication and OTU clustering using VSEARCH, and taxonomic assignment by BLASTn with a 90% minimum consensus threshold using the Greengenes and SILVA 99% databases. B. Taxonomic classifications obtained from *k*-mer based indexing implemented by Kraken 2.

**Additional file 2: Supplemental Figure S1**: A. The increased detection rate in WGS data compared to 16S is maintained at full coverage. B. The detected members of each predominant phylogenetic group at any level. The highest membership across the four predominant phyla in 16S (red) and WGS (blue) was identified in WGS at full coverage. **Supplemental Figure S2**: The alpha diversity rarefaction of the 16S data set shows the distribution of Fath’s Phylogenetic Diversity (Faith’s PD) index over increasing sequencing depths. The high protein, low carbohydrate diet (HPLC) shows a diminished community richness compared to the baseline diet (BASE) and low protein, high carbohydrate (LPHC) diets, which share similar distributions of phylogenetic diversity at higher sequencing depths. **Supplemental Figure S3**: A. The beta diversity (Bray-Curtis Dissimilarity) metric multidimensional scaling reveals dissimilarities in the compositional differences of high protein, low carbohydrate (HPLC) and low protein, high carbohydrate (LPHC) WGS microbial communities, and the most dissimilar communities have a higher abundance of *B. coprocola*. B. A similar analysis of WGS gene family communities indicates HPLC and LPHC diets have greater dissimilarity. These dissimilar communities have a higher *LeuB* relative abundance, which is coenriched with *B. coprocola*. **Supplemental Figure S4**: According to the performance metrics for 12 classification algorithms, the random forest achieves the highest overall percent accuracy in classifying WGS samples. The random forest ties with the gradient boosted regression tree for the highest percent accuracy in classifying 16S samples and achieves the lowest log loss.

## Data Availability

All data analyzed during this study are included in these published articles: [9] for 16S dataset (NCBI Sequence Read Archive accession number SRP095473) and [10] for WGS dataset (European Nucleotide Archive accession number PRJEB20308).

## Abbreviations

HPLC: High protein, low carbohydrate diet
LPHC: Low protein, high carbohydrate diet
OW: Overweight body fat phenotype
LN: Lean body fat phenotype
WGS: Whole genome shotgun sequencing
16S: 16S amplicon sequencing
OTU: Operational taxonomic unit
ASV: Amplicon sequence variant
Faith’s PD: Faith’s Phylogenetic Diversity index
RAxML: Randomized Axelerated Maximum Likelihood algorithm
LDA: Linear discriminant analysis
SVM: Support vector machine
SVD: Singular value decomposition
AUC: Area under the curve
ROC: Receiver operating characteristic
