# Supplementary material for: Comparison of 16S and whole genome dog microbiomes using machine learning
Source: BioData Min. 2021 Aug 21;14:41. doi: 10.1186/s13040-021-00270-x (PMC8379800; doi:10.1186/s13040-021-00270-x)
Supplement: Supplementary file 1 [file 13040_2021_270_MOESM1_ESM.docx]

Supp. Table 1

A

	Species	Bacteroidetes	Firmicutes	Proteobacteria	Actinobacteria
GreenGenes	25	20	63	25	11
SILVA	28	29	175	33	14

B

	Species	Bacteroidetes	Firmicutes	Proteobacteria	Actinobacteria
Kraken 2	3247	622	1153	3449	1242
