# Supplementary material for: Comparison of 16S and whole genome dog microbiomes using machine learning
Source: BioData Min. 2021 Aug 21;14:41. doi: 10.1186/s13040-021-00270-x (PMC8379800; doi:10.1186/s13040-021-00270-x)

Supp. Figure S1 (16S Vs. Full Coverage WGS)

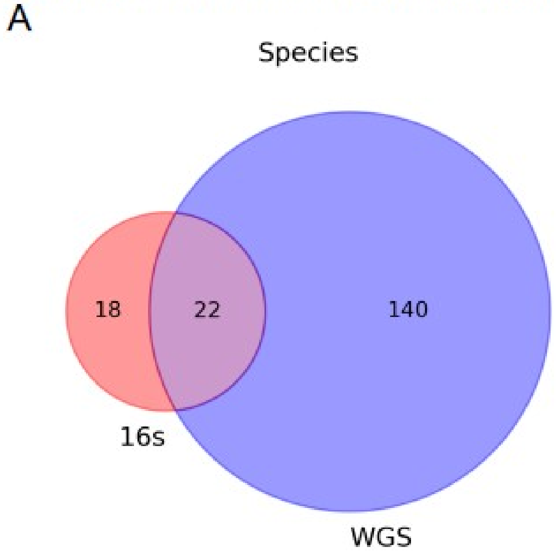

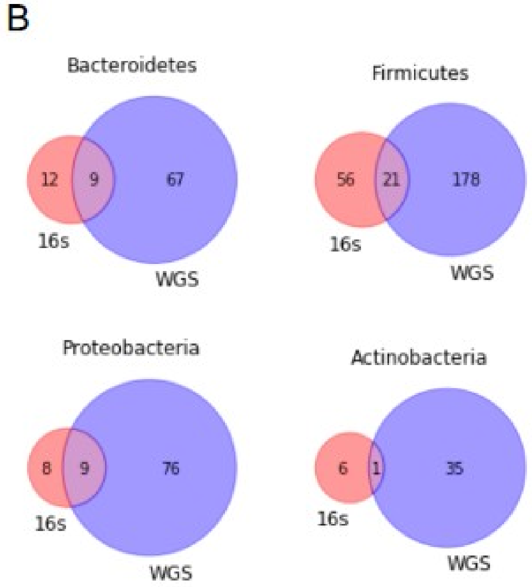

Supp. Figure S2

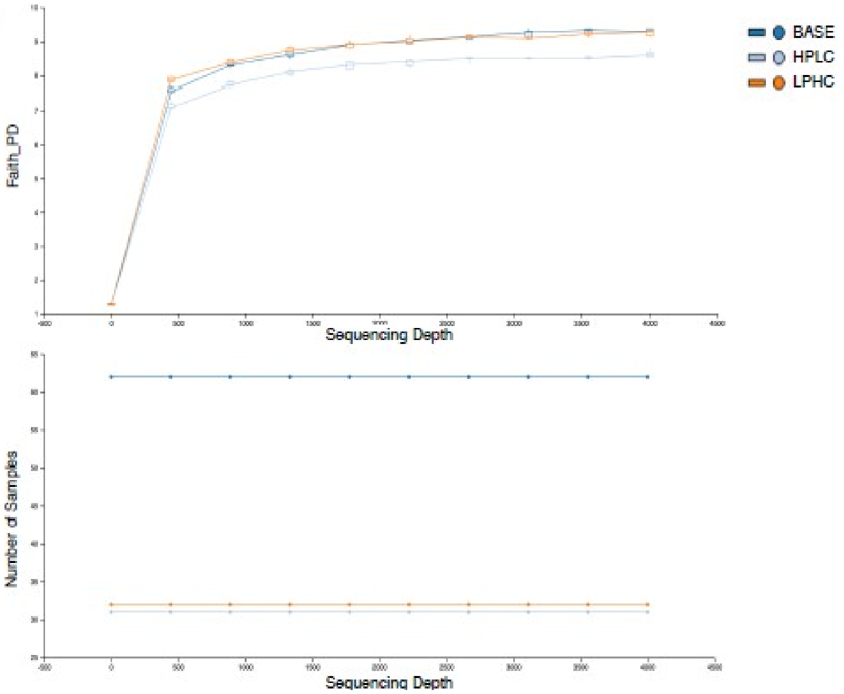

Supp. Figure S3

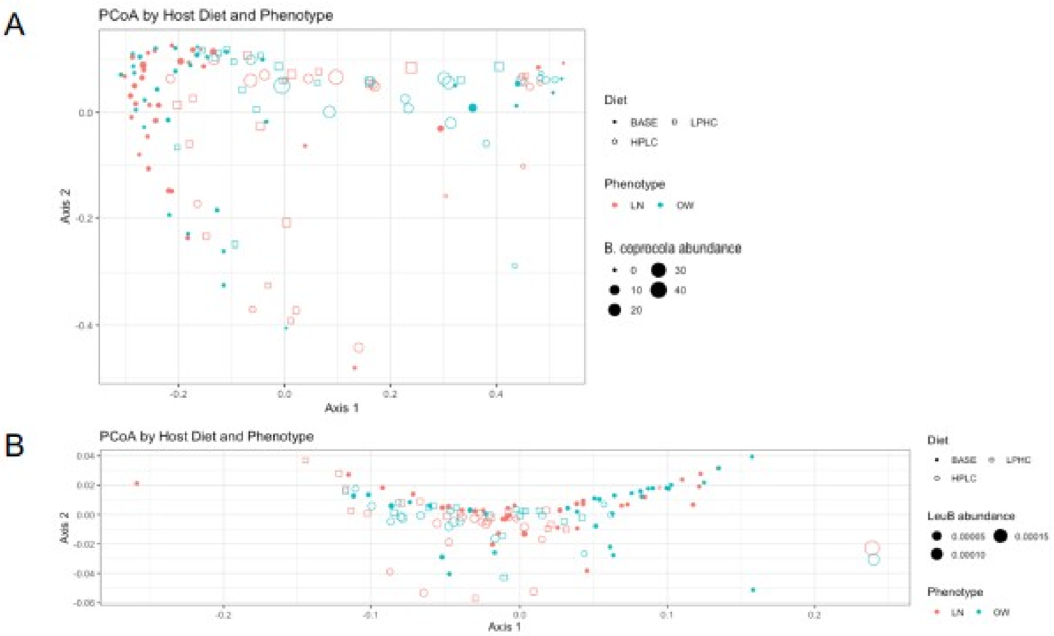

Supp. Figure S4

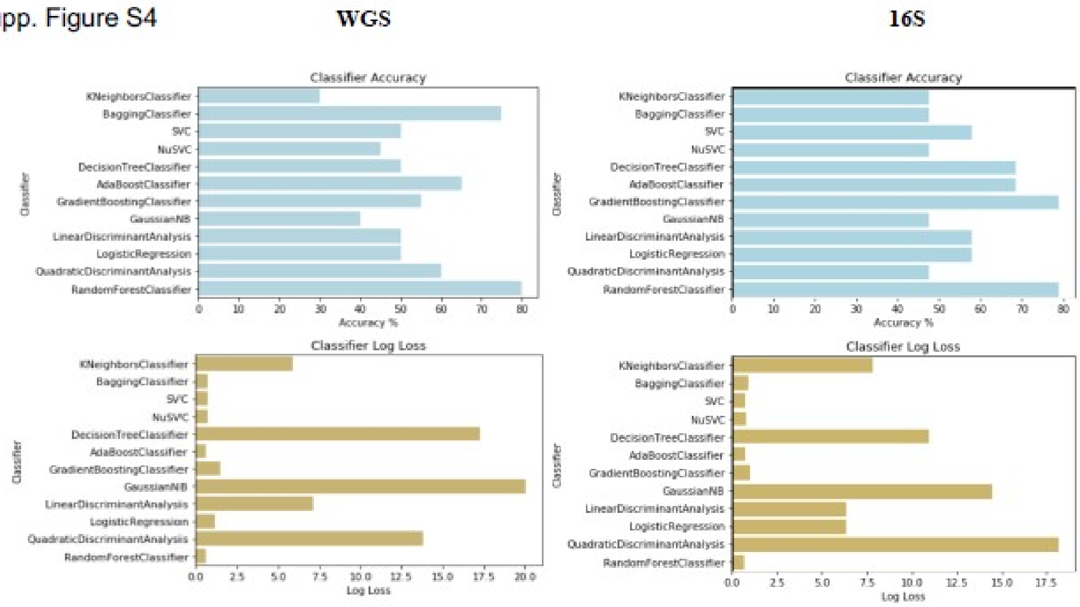

Supplement: Supplementary file 2 [file 13040_2021_270_MOESM2_ESM.docx]
